# SCM: a practical tool to implement hospital-based syndromic surveillance

**DOI:** 10.1186/s13104-016-2098-z

**Published:** 2016-06-18

**Authors:** Chuchu Ye, Zhongjie Li, Yifei Fu, Yajia Lan, Weiping Zhu, Dinglun Zhou, Honglong Zhang, Shengjie Lai, David L. Buckeridge, Qiao Sun, Weizhong Yang

**Affiliations:** Research Base of Key Laboratory of Surveillance and Early-warning on Infectious Disease in China CDC, Shanghai Pudong New Area Center for Disease Control and Prevention, Shanghai, China; Key Laboratory of Surveillance and Early-warning on Infectious Disease, Chinese Center for Disease Control and Prevention, Beijing, China; Research Base of Key Laboratory of Surveillance and Early-warning on Infectious Disease in China CDC, West China School of Public Health, Sichuan University, Chengdu, China; Department of Geography and Environment, University of Southampton, Southampton, UK; McGill University, Montreal, Canada

**Keywords:** Syndromic surveillance, Infectious disease, Mass gatherings, Early warning, Outbreak detection

## Abstract

**Background:**

Syndromic surveillance has been widely used for the early warning of infectious disease outbreaks, especially in mass gatherings, but the collection of electronic data on symptoms in hospitals is one of the fundamental challenges that must be overcome during operating a syndromic surveillance system. The objective of our study is to describe and evaluate the implementation of a symptom-clicking-module (SCM) as a part of the enhanced hospital-based syndromic surveillance during the 41st World Exposition in Shanghai, China, 2010.

**Methods:**

The SCM, including 25 targeted symptoms, was embedded in the sentinels’ Hospital Information Systems (HIS). The clinicians used SCM to record these information of all the visiting patients, and data were collated and transmitted automatically in daily batches. The symptoms were categorized into seven targeted syndromes using pre-defined criteria, and statistical algorithms were applied to detect temporal aberrations in the data series.

**Results:**

SCM was deployed successfully in each sentinel hospital and was operated during the 184-day surveillance period. A total of 1,730,797 patient encounters were recorded by SCM, and 6.1 % (105,352 visits) met the criteria of the seven targeted syndromes. Acute respiratory and gastrointestinal syndromes were reported most frequently, accounted for 92.1 % of reports in all syndromes, and the aggregated time-series presented an obvious day-of-week variation over the study period. In total, 191 aberration signals were triggered, and none of them were identified as outbreaks after verification and field investigation.

**Conclusions:**

SCM has acted as a practical tool for recording symptoms in the hospital-based enhanced syndromic surveillance system during the 41st World Exposition in Shanghai, in the context of without a preexisting electronic tool to collect syndromic data in the HIS of the sentinel hospitals.

## Background

Surveillance systems play a fundamental role on the monitoring and detecting outbreaks of infectious diseases. Syndromic surveillance, or the use of near “real-time” pre-diagnostic data and automated tools to detect and characterize unusual activity for further public health investigation, has been adopted in many countries to augment traditional surveillance for nearly two decades [1–3]. Syndromic surveillance systems usually employ statistical algorithms to inspect unexpected changes in prodromic or pre-diagnostic data captured in electronic systems from a variety of sources, with the premise that aberrations in these data may provide an earlier indication of a disease outbreak before the change can be observed in confirmed diagnoses [4, 5]. Among the multiple sources of pre-diagnostic data (such as emergency department visits, ambulance trip logs, pharmacy sales, and work or school absentee rates), pre-diagnostic clinical syndromes among patients visiting hospitals are frequently used as a data source in syndromic surveillance systems [6–8]. As syndromic surveillance is expected to detect an epidemic at a very early stage, the timely collection, collation and analysis of a large amount of syndrome data are key system components.

In most existing syndromic surveillance systems, a common strategy of obtaining data from hospitals is to automatically collect the chief complaints of patients and to classify the data into syndromic categories using standard codes or by natural language processing [9–11].Syndromic surveillance, however, faces huge challenge while the chief complaints have not been documented electronically in the hospitals, which had been encountered in Pudong New Area, Shanghai. The most of the hospitals in Pudong have an electric hospital information system (HIS) to record medical information. When a patient visits a hospital, a social insurance card or a provisional electronic card must be presented at the reception counter. Basic demographic information about the patient is recorded in the HIS, such as gender, date of birth, address and historical medical record. The HIS then generates a patient ID number, which is unique for the visit, and the patient is subsequently seen by a doctor. Information on the patient can be tracked by the ID number, which can be used to access the HIS record, including lab test orders, test results, prescriptions, and diagnoses. However, the fundamental information for syndromic surveillance-the chief complaints of the patient, are not routinely collected in HIS, as it is practically recorded on paper at the stage of triage.

The 41st World Exposition was held in Shanghai city, China, from May 1st to October 31st, 2010. It has been the largest exposition in the history, with more than 73 million visitors from approximately 240 countries and organizations. A large flow of international visitors might pose a potential public health risk of disease importation, as infectious disease transmission can be promoted by an increasing population flow and density or by the accidental or deliberate introduction of unusual pathogens. Pudong New Area is the largest district of Shanghai city, containing 5.4 million residents and approximately 60 % areas of the World Exposition sites. A routine notifiable infectious disease reporting system has been used, which only collected information of specific kinds of infectious disease diagnosed by the clinicians. To enhance the sensitivity and timeliness of disease outbreaks detection during the Expo, a syndromic surveillance and early warning system, Pudong Syndromic surveillance and Early Warning System (PD-SEWS), was implemented in this district.

To allow automated electronic access to chief complaints for PD-SEWS in the 41st World Exposition held in Shanghai in 2010, we have piloted to introduce a novel approach, the symptom-clicking-module (SCM), to gather syndrome data from hospitals, thereby enabling implementation of syndromic surveillance. Here, we describe how SCM operated, and present the main results of targeted syndromes, and compare the patterns of data among different levels of hospitals, as well as the day-of-week effect.

## Methods

### Surveillance sentinels and targeted syndromes

**Fig. 1:**
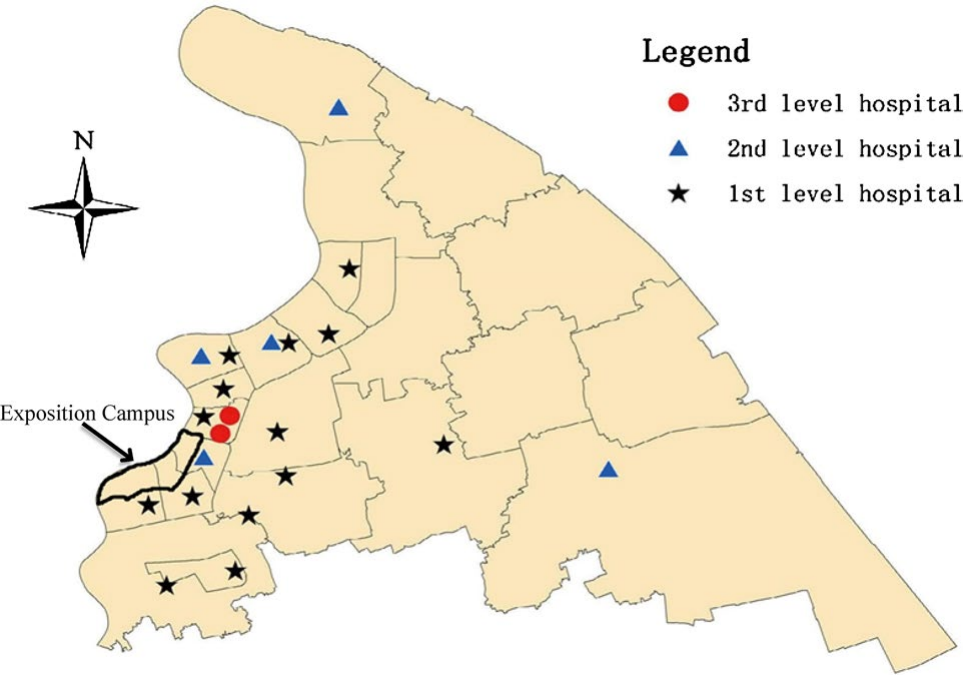
The geographic location of sentinel hospitals at three different levels in Pudong New Area, Shanghai, China, 2010

Twenty-one hospitals were selected as sentinel sites for PD-SEWS, including two tertiary hospitals, five secondary hospitals and fourteen primary hospitals. Hospitals were chosen according to their location, catchment area, and patient volume [12]. In Pudong, a primary hospital is the smallest category of healthcare facility, providing primary medical services such as medical treatment, prevention, healthcare, and rehabilitation for a community with a population of <100,000 persons. The secondary hospitals provide general medical services, including medical treatment, prevention, healthcare, and rehabilitation for larger communities (population >100,000 persons). The tertiary hospitals are regional healthcare centers providing specialized, high-complexity healthcare services for several districts. Most of the sentinel sites were closed to the Exposition venue, where was also the part of Pudong with highest population density (Fig. 1).

The concerning diseases during mass gatherings and their typical symptoms were listed according to literature review, and Delphi method was employed to consult 18 domestic epidemiologic and clinical professionals to score the disease severity, risk probabilities. Then a disease-risk matrix was draw and 40 diseases were prioritized in the surveillance, including local common diseases and some highly concerned diseases of importation. The 25 most common and typical symptoms of the targeted diseases were enrolled and classified to seven targeted syndromes: acute respiratory, acute gastrointestinal, rash with fever, neurological syndrome, hemorrhagic fever, botulism-like syndrome and acute viral hepatitis (Table 1).

**Table 1:** The seven syndromes under surveillance in the 41st exposition, Pudong New Area, Shanghai City, China, 2010

Syndrome	Typical symptoms
Acute respiratory syndrome	Fever with at least one of the following: cough, sputum, hemoptysis, chest pain, breathing difficulties
Acute gastrointestinal syndrome	Fever with at least one of the following: vomiting, diarrhea, pus/mucus in stool
Rash with fever	Fever with at least one of the following: herpes, maculopapular rash
Neurological syndrome	Fever with at least one of the following: headache, projectile vomiting, shock, altered consciousness, sudden body pain
Hemorrhagic fever	Fever with at least one of the following: skin or mucous congestion, petechiae, bleeding, bloody stool
Botulism-like syndrome	At least one of the following: sudden blurred vision, dysphagia
Acute viral hepatitis	At least one of the following: hepatosplenomegaly, acute jaundice, lymphadenopathy

### Data collection and transmission

**Fig. 2:**
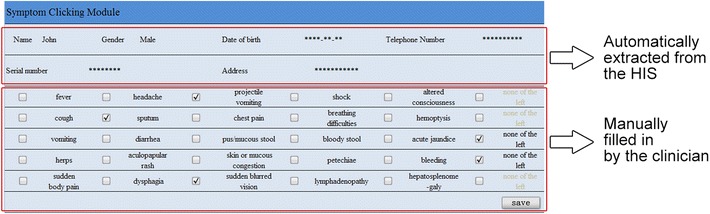
User interface of the symptom-clicking-module of PD-SEWS, Shanghai, China, 2010. All English words in the SCM were translated from Chinese words

**Fig. 3:**
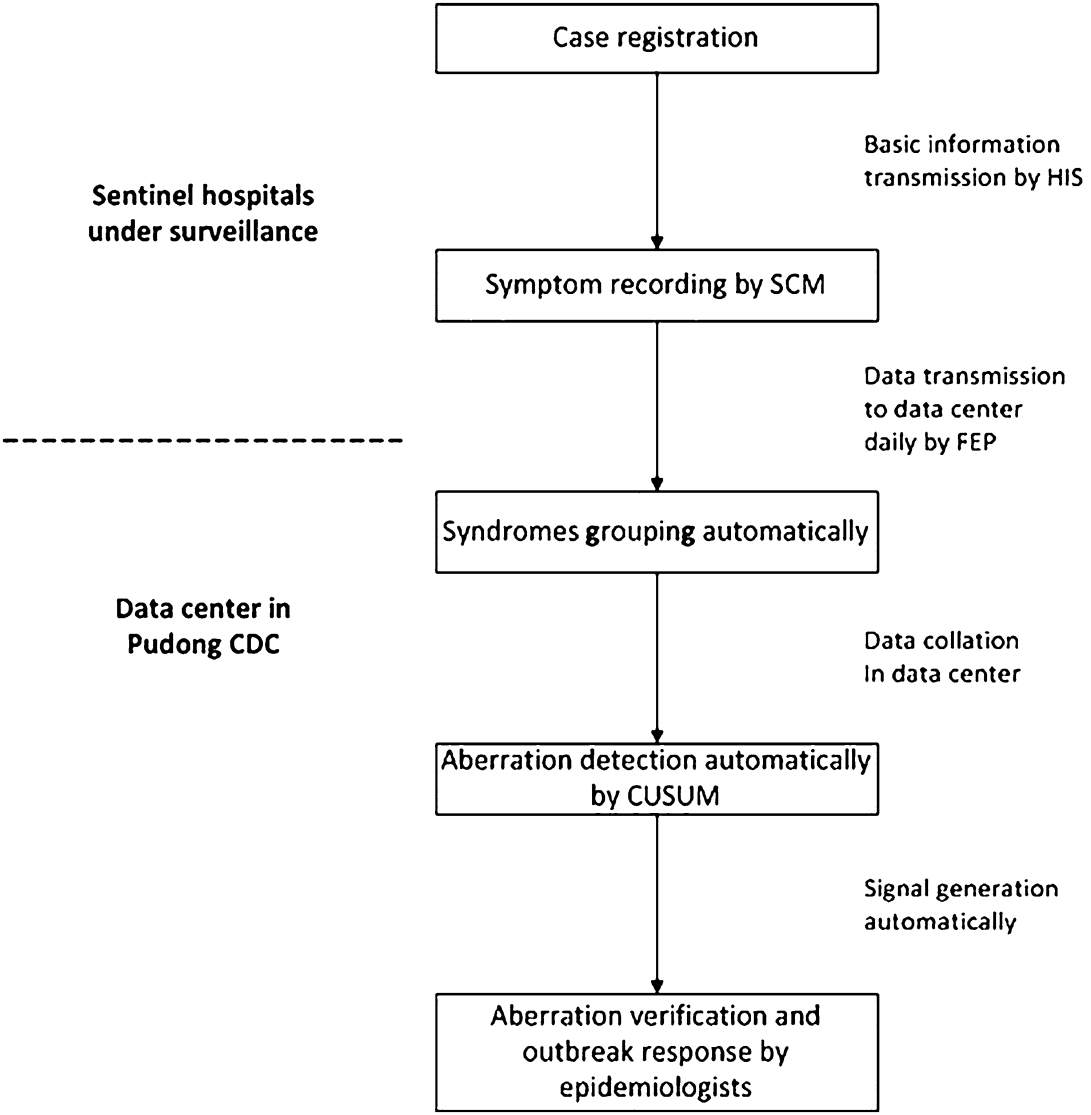
Framework of syndromic surveillance in Pudong New Area, Shanghai, China, 2010

The SCM was developed and embedded in the HIS for each sentinel hospital, with the 25 symptoms presented on the SCM interface as a 5 × 5 table which could be selected by single click on the screen (Fig. 2). In addition, we included an extra column to facilitate data entry and improve data quality. If no symptoms in a row were presented, the ‘none of the left’ could be clicked. A clinician could therefore check each row of the table quickly. For example, if a patient described the chief complaint as ‘fever and cough’, the clinician should click ‘fever’ in the first row and ‘cough’ in the second, as well as ‘none of the left’ for the other three rows. Clinicians were prevented from moving to the next step of treatment (such as prescribing a medication) until they finished recording symptoms by clicking the ‘save’ button (Fig. 3).

A record of symptoms in SCM was generated with a unique identity (ID) number and the basic demographic information registered in HIS. All data were stored in real time and transmitted automatically to the Pudong Public Health Database Center each day. A virtual private network (VPN) was used to connect securely with sentinel hospitals, and all data were supplied and analyzed in an anonymous format, without access to personal identifying information. For improving the data quality, duplicated records were rejected by checking the unique ID number, and if the data were not received from a sentinel site, the Public Health Database Center would send a notice to that hospital at 08:00 a.m. the next day. Once the database center received the surveillance data, all of the patient records were automatically grouped and aggregated into the seven syndromes according to the criteria listed in Table 1. If one patient’s symptoms referred to more than one syndrome, then the encounter would be counted separately for each syndrome.

### Data analysis and aberration detection

We developed an interface connecting to the Pudong Public Health Database Center. The interface allowed authorized users to generate customized time series of total visits for each syndrome stratified by gender, age, syndrome, hospital, start dates and end dates. The cumulative sum (CUSUM) method, a control chart method commonly used in syndromic surveillance [4, 13], was applied daily to analyze the aggregated data of all sentinel hospitals for detecting abnormal temporal increases [14]. For each targeted syndrome, CUSUM compared the proportion of syndrome counts in total visits in the current day (day 0) with the corresponding mean proportion and standard deviation of the past 7 days (day-7 to day-1). A signal was generated if the value of comparison exceeded two standard deviations.

The surveillance and response team of Pudong Center for Disease Control and prevention (Pudong CDC) would monitor the warning signals routinely. When a signal was triggered, the verification would be conducted immediately by analyzing and reviewing the data to identify any unusual cluster of gender, age, occupation or hospital. Some signals would be verified and compared with the data in the routine notifiable disease reporting system, which recorded the confirmed patients’ detailed information. Potential epidemic association would be explored by calling the clinicians or patients. If the signal indicated a potential outbreak, field investigation would be performed to obtain more detailed epidemiological information and necessary control measures would be conducted to prevent further spread of the disease. Outbreak is defined as the occurrence of cases of disease in excess of what would normally be expected in a defined geographical area and period, which was further quantitatively defined for each kind of disease in this study, so as to facilitate the outbreak confirmation and report. For example, an outbreak of hand, foot and mouth (HFM) disease is defined as “within 1 week, at least five HFM disease cases occur in the same setting-e.g. kindergarten or school-or at least three cases of the disease occur in the same village or community” [15].

In this study, the number of outpatients, the reporting frequency and the proportion of each syndrome were calculated. The average reporting frequencies of different syndromes between weekdays and weekends were compared using a Chi square test, and a *P* value <0.05 was considered to be statistically significant.

### Preparation for system operation

A series of training sessions on the system were conducted for the clinicians in the sentinel hospitals before the practical operation. This approach helped to ensure that the clinicians would be familiar with SCM in HIS. The estimated average time cost of recording a patient’s syndrome by SCM was 10 s, according to our field investigation in the last week of the training. A pilot surveillance operation was conducted 2 weeks before the formal running of this syndromic surveillance system, and the staff from Pudong CDC went to all the 21 sentinel hospitals during this pilot period to provide help if needed.

## Results

During the surveillance period (184 days) from May 1, 2010 to October 31, 2010, totally 1,730,797 patient encounters were collected through the SCM. Of these encounters, 105,352 (6.1 %) were classified into the seven targeted syndromes under surveillance. The most frequent syndromes were acute respiratory syndrome (59,793 encounters) and acute gastrointestinal syndrome (45,634 encounters), which together accounted for 92.1 % of total visitors in the seven targeted syndromes. Botulism-like syndrome and hemorrhagic fever syndrome had the lowest frequencies, with only 187 and 131 encounters, respectively. Encounters classified as acute respiratory, acute gastrointestinal, and neurological syndromes were reported every day, whereas the other syndromes (including rash with fever, acute viral hepatitis, botulism-like syndrome and hemorrhagic fever) were not found from 7 to 122 days during surveillance period (Table 2).

**Table 2:** Descriptive statistics of visit counts for the seven syndromes in PD-SEWS, Shanghai, China, May 1st to Oct 31st, 2010

Syndrome	Overall visits	Proportion of the total visits (%)	Mean visits per day	Minimum visits per day	Q1 visits per day	Median visits per day	Q3 visits per day	Maximum visits per day	Days with zero reporting
Acute respiratory syndrome	59,793	3.45	325	143	273	310.5	356.5	642	0
Acute gastrointestinal syndrome	45,634	2.64	248	123	196.8	249	295.8	398	0
Neurological syndrome	6055	0.35	32.9	9	20	29	41	112	0
Rash with fever	1910	0.11	10.4	0	3.8	7.5	16	40	7
Acute viral hepatitis	790	0.05	4.3	0	1	3	6	26	25
Botulism-like syndrome	187	0.01	1	0	0	0	2	13	110
Hemorrhagic fever	131	0.01	0.7	0	0	0	1	7	122

*Q1* first quartile value; *Q3* third quartile value

**Fig. 4:**
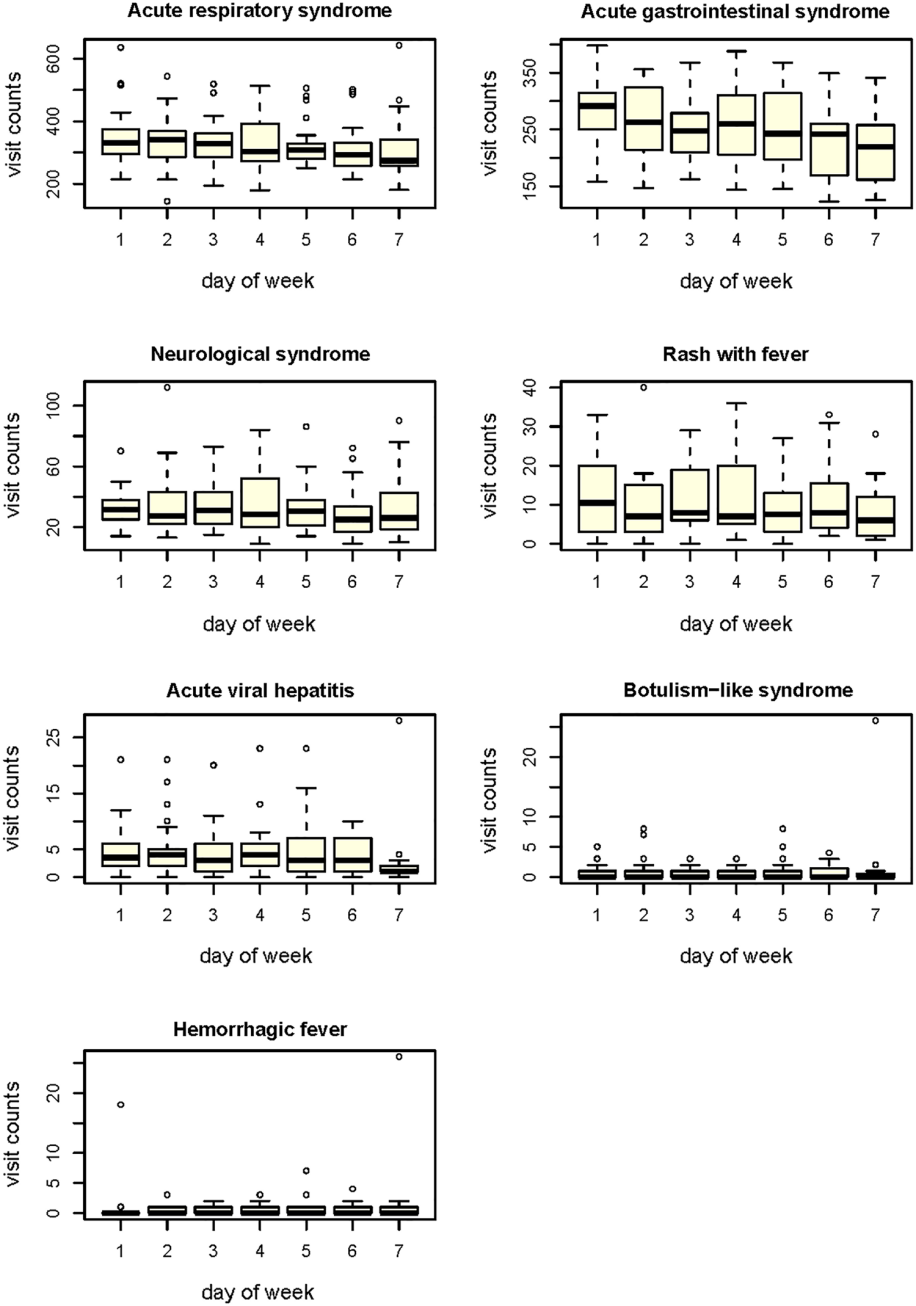
*Box plot* of average reporting number of each day of week by syndrome detected by PD-SEWS, Shanghai, China, 2010

The reporting frequencies for different syndromes varied with the day of the week. Except for hemorrhagic fever, the frequency of all other six surveillance syndromes were higher on the weekdays than that in weekends, but the variation was only significant for acute gastrointestinal syndrome and acute viral hepatitis (Fig. 4). Additionally, in terms of the average reporting frequency of each syndrome across the sentinel hospitals, we found that the tertiary and secondary hospitals overall contributed much more than the primary hospitals, and the differences varied with syndromes. For rash with fever syndrome, the average reporting number in the tertiary hospitals was 151 times of that in the primary ones. However, for acute viral hepatitis syndrome, the average reporting numbers in the three levels of hospital were closer (Table 3).

**Table 3:** Average reporting amount of each syndrome per hospital by levels of hospital in Pudong New Area, Shanghai, China, 2010

Syndromes	Average reporting amount by hospital’s level			Ratio of primary to secondary and tertiary hospital (a:b:c)
	Primary (a)	Secondary (b)	Tertiary (c)	
Acute respiratory syndrome	707.4	8544.0	3585.0	1:12:5
Acute gastrointestinal syndrome	756.0	5508.8	3753.0	1:7:5
Neurological syndrome	63.7	920.6	280.0	1:14:4
Rash with fever	4.4	106.4	658.5	1:24:151
Acute viral hepatitis	28.4	56.8	54.5	1:2:2
Botulism-like syndrome	3.3	15.0	33.0	1:5:10
Hemorrhagic fever	3.0	9.2	21.5	1:3:7

During the surveillance period, the CUSUM triggered 191 signals, including 44 signals for acute respiratory syndrome, 41 signals for neurological syndrome, and 30 signals for acute gastrointestinal syndrome, and rash with fever syndrome, botulism-like syndrome, hemorrhagic fever and acute viral hepatitis had 22, 22, 17 and 15 signals respectively. All of the signals had been verified, and field investigation and control measures had been conducted if a signal seemed to lead to an outbreak. Finally, none of the signals were confirmed as outbreaks. Meanwhile, there was no infectious disease outbreak or public health emergency was confirmed and reported by local CDC through the other routine surveillance system during the same period.

## Discussion

The SCM was implemented successfully and operated for a surveillance period lasting 184 days in Expo 2010, which made it feasible to collect syndromic data and classify syndromes which was lacking in the HIS of sentinel hospitals. Approximately 6 % of patient encounters were classified into the seven surveillance syndromes, with the number of patients ranged from 131 in hemorrhagic fever syndrome to 59,793 in acute respiratory syndrome. Frequency of reporting varied by days of week for some syndromes, and the secondary and tertiary hospitals reported the majority of encounters in syndrome surveillance.

In recent years, enhanced surveillance is commonly implemented during mass gatherings [16], and syndromic surveillance system has been established globally. In some countries, more efforts are needed to improve data collection to make the establishment of syndromic surveillance more easily and cost-effectively [17, 18]. The SCM in this study collated and analyzed syndromic data in a standard format across reporting hospitals, and allowed direct and automatic classification of symptoms into syndromes, avoiding to develop additional syndrome classification software [19, 20]. Instead of directly collecting the syndrome data subjectively decided by the clinicians, SCM allowed clinicians to record the raw data of symptoms on a patient, and the system automatically grouped the encounters into corresponding surveillance syndromes using pre-defined syndrome definitions. Additionally, SCM was embedded into the HIS so that all the basic information of the patients could be extracted from the registration data collected automatically. The design of SCM saves time during data collection and helps to avoid errors due to manual syndrome classification, which is important and effective because the most existing HIS in China without recording chief complaints of patients.

We found that acute respiratory and acute gastrointestinal syndromes were reported most frequently, which is consistent with other syndromic surveillance systems [21–23]. These syndromes cover the symptoms of some common diseases, such as influenza and viral diarrhea, which are also the main infectious diseases in Shanghai during the study period. Moreover, the day-of-week effect in our data suggests the residents in study area avoided to seek healthcare during weekend, possibly because hospitals could provide more medical services on weekdays. Therefore, the aberration detection algorithm on the syndromic surveillance system should take account of the day-of-week effect [22].

The variation in reporting across the different levels of hospital was also observed for each syndrome. We found that most of the fever with rash cases were reported by one tertiary hospital, which was the biggest children’s hospital in Pudong. Fever with rash syndrome usually presented among the diseases such as measles, rubella or chicken pox with high incidences in children. The primary hospitals reported the least cases in all of the syndromes, but the difference seemed much smaller for the acute viral hepatitis syndrome. This difference is probably because most of the chronic hepatitis patients sought medical services at the nearest primary hospital. Additionally, the SCM is a new data collection module embedded into the routine HIS at each hospital, and the clinicians might need some time to accustom his change in their routine workflow. The strategy of conducting pilot surveillance is widely adopted by syndromic surveillance practice during mass gathering, which is key to improve the system’s acceptability and ensure the data quality, which has been also conducted in this study [22–24].

In this study, no one outbreak was confirmed among the 191 signals generated by PD-SEWS during the Expo. Each signal, which represented an aberration on the surveillance data, was required to be timely verified and investigated by the local staff of Center for Disease Control and Prevention, and control measures should be conducted rapidly once a signal seemed to lead to an outbreak. That’s the possible reason why no one signal was identified as an outbreak finally. To enhance the routine surveillance system by increasing the sensitive and timely detection of outbreaks was the important objective of syndromic surveillance, as well as to deal with each signal before it led to a real outbreak. The system would create a lot of extra work for Public Health Utility, that’s why we only suggested adopting the enhanced syndromic surveillance system during the specific period, such as holding mass gatherings, instead of conducting it as routine work after the Expo.

There were some limitations in our study. First, the cost-effectiveness of the SCM application was not evaluated systematically, but we performed a pilot investigation of the SCM’s usability and acceptability in several hospitals at the beginning of the surveillance. In addition, we have not systematically collected and evaluation the feedbacks from the clinicians during the study period. Nonetheless, according to the data collected by SCM and its operating results, it sounds acceptable for physicians to use during the special situation of a mass gathering. Another limitation is that spatial cluster detection was not conducted in our study. Some algorithms such as space–time scan statistic [25] have been applied to detect spatial aberration, but SCM without the sufficient geographic information of each patient to conduct this analysis. Additionally, as the CUSUM method was applied to the aggregated data of all hospitals instead of each single sentinel site, this limited the ability to detect small outbreaks. However, as the surveillance data for each sentinel hospital (including three different levels of hospitals) were not stable enough on the time series, which would lead to large amount of false alerts if performing the algorithms on single hospital level. Therefore, taking account of the balance between sensitivity and false alarm rate, we applied CUSUM method to the total number of each syndrome, and adopted a relatively lower threshold to ensure the sensitivity. In addition, the system could automatically demonstrate daily time series figure of surveillance data for each single hospital, which could assist in the local epidemiologist to detect the potential smaller outbreak with manual manner.

## Conclusions

SCM has acted as a practical tool for recording symptoms in the hospital-based enhanced syndromic surveillance system during the 41st World Exposition in Shanghai, in the context of without a preexisting electronic tool to collect syndromic data in the HIS of the sentinel hospitals.

## Abbreviations

SCM: symptom-clicking-module
HIS: hospital information system
PD-SEWS: pudong syndromic surveillance and early warning system
FEP: front end processor
PDCDC: Pudong New Area Center for disease control and prevention
VPN: virtual private network
CUSUM: cumulative sum
